# The Case against Forced Methadone Detox in the US Prisons

**DOI:** 10.1093/phe/phw040

**Published:** 2016-11-19

**Authors:** Daniel D’Hotman, Jonathan Pugh, Thomas Douglas

**Affiliations:** 1 Oxford Uehiro Centre for Practical Ethics, University of Oxford; 2 Faculty of Medicine, Nursing and Health Sciences, Monash University

## Abstract

Methadone maintenance therapy is a cost-effective, evidence-based treatment for heroin dependence. In the USA, a majority of heroin-dependent offenders are forced to detox from methadone when incarcerated. Recent research published in *The Lancet* has demonstrated the negative health and economic outcomes associated with such policies (Rich, J. D., McKenzie, M., Larney, S., Wong, J. B., Tran, L., Clarke, J. *et al.* (2015). Methadone Continuation Versus Forced Withdrawal on Incarceration in a Combined US Prison and Jail: A Randomised, Open Label Trial. *The Lancet*, 386, 350–359). This novel evidence raises questions as to the justification for current policies of forced detox in American prisons. Opponents of methadone provision in prisons might offer arguments from retributivism, resource allocation and curative effectiveness to justify their position. This article contends that these arguments do not stand up to ethical scrutiny. In light of this, we hold that American policymakers should reform criminal justice policies to allow the initiation and continuation of methadone treatment in correctional settings. This would be consistent with both international recommendations and the example set by a number of other Western countries.

## Introduction

Methadone is a long-acting opioid used for the treatment of opiate addiction in what is termed methadone maintenance therapy (MMT). Due to its activation of similar neural pathways to heroin, methadone may be used as a substitution treatment for heroin addicts to prevent withdrawal, block the euphoric effects of heroin and relieve narcotic cravings (Joseph et al., 2000). Although these effects are dose dependent, and vary between patients, an effective dose results in a decreased desire to consume heroin. In addition, methadone is non-sedating and non-euphoric at stable doses, meaning that patients are able to work, drive and feel a full range of emotions without narcotic impairment (Joseph et al., 2000).

Research published by Rich *et al.* in the May 2015 issue of *The Lancet* provides insight into the harm caused by forced methadone detox in the US correctional facilities. This practice involves requiring heroin-dependent persons who were receiving methadone treatment in the community to cease taking the drug upon incarceration. Rich *et al.* found that heroin-dependent prisoners who continued methadone treatment following their incarceration were seven times more likely to engage in methadone treatment after release than prisoners in the forced detox group. Furthermore, continued methadone use resulted in fewer medical costs in the month after release, and reduced societal costs by $1632 per person when compared with those required to cease taking methadone. Despite this, more than 90 per cent of incarcerated offenders on prescribed methadone are required to detox in the US correctional facilities every year (Rich et al., 2015).

## The Case for Continuing MMT in Prisons

Like other countries, the USA has a strong public health interest in the effective treatment of heroin dependence. The transmission of HIV and other blood-borne infections is a significant problem amongst intravenous heroin users, and drug use often continues in prison (Lawrinson et al., 2008). Prisoners who inject drugs are more likely than non-users to contract HIV and hepatitis, as well as transmitting these infections to other inmates through needle-sharing and high-risk sexual activities (Mattick et al., 2009). Public health authorities should aim to reduce spread of these dangerous infections, and evidence demonstrates that MMT is a feasible, safe and cost-effective way to achieve this goal (Lawrinson et al., 2008).

In addition, violent crime might itself be regarded as a public threat in light of the health risk it poses to others, and heroin addiction is a known risk factor for violent offending (Boles and Miotto, 2003; Tomison, 2009). MMT has been shown to be an effective means of mitigating this risk (Rich et al., 2005; Mattick et al., 2009). One Australian study found that for every 100 persons in a MMT programme, even when adjusting for other factors, there were 22 fewer criminal charges filed in the judicial system per year (Lind et al., 2005). Moreover, these results may understate MMT’s positive effect on criminal activity, as it is impossible to estimate the many crimes never reported to police, and only 46 per cent of violent crimes and 16 per cent of property crimes reported actually result in criminal charges (Harrison, 2001).

There is thus a strong public health case for continuing MMT during periods of incarceration. Prisoners have a long-established right to a reasonable standard of healthcare that is comparable to that of other members of the community. For example, it is widely accepted that offenders with chronic medical conditions such as heart disease, hypertension or AIDS retain the right to continue their medications when they have been convicted of a crime. Yet, despite a significant body of research indicating that substance dependence is a chronic, ongoing disorder, opioid-addicted offenders in most American states do not receive the treatment they might receive if they were in a typical community setting. Admittedly, some prisoners in the US prisons have access to a range of psychosocial interventions (cognitive behavioural therapy and counselling); however, these interventions are rarely effective as a sole treatment for heroin dependence in prisons where they are available. A Cochrane review of 11 randomized control trials, including three trials conducted on prisoners, demonstrated that MMT was more effective in retaining patients and suppressing heroin use, and received better reviews in patient self-reports than drug-free alternatives^1^ (Mattick et al., 2009). As a result, preventing prisoners from accessing methadone treatment seems inconsistent with the principle of evidence-based practice that is upheld in the drug treatment community.

In view of the clinical and public health arguments in its favour, many Western countries, including Australia, Canada and all European member nations (Farrell et al., 2000; [Bibr phw040-B24][Bibr phw040-B16]; ), have made MMT available in correctional facilities. Both the United Nations and World Health Organization recommend that offenders with substance dependence should be issued with treatment rather than punishment (Joint United Nations Programme on HIV/AIDS \(UNAIDS\), 2004; World Health Organisation, 2008; Arria et al., 2009). However, the USA continues to reject these recommendations, as well as the policy example set by other Western nations. In 2007, 11.4 per cent of Australian inmates received opiate replacement therapy, while the same was true for less than 0.1 per cent of their American counterparts (Larney, 2011).

## Justifications for Forced Methadone Detox

The US reluctance to embrace MMT in prisons and preference for detoxification may be due in a large part to opposition of prison workers, judges and politicians. For example, a 2005 survey of prison directors in 50 states found that only 48 per cent supported the use of methadone in their prisons, and only 8 per cent referred heroin-dependent prisoners to methadone therapy after release (Rich et al., 2005). Can this reluctance be justified? A number of arguments might be offered in defence of the US approach:

1. There has been concern that the presence of methadone in prison settings would lead to the proliferation of a ‘black market’ for the drug. However, there is evidence that the risk of drug diversion can be significantly reduced through the implementation of properly supervised dispensing practices; for example, nursing staff could dispense methadone as well as supervising prisoners to ensure compliance and reduce costs (Gowing et al., 2014). Moreover, multiple reports have demonstrated that the trade of illegal drugs is already rampant in the US prisons (Fazel et al., 2006). It is not clear why the addition of methadone to this market would exacerbate the problem, and indeed to the extent that methadone availability displaces demand for more addictive and risky substances (i.e. heroin), its presence in the market might be regarded as the lesser evil.
2. It might be argued that criminal offenders must be punished for their wrongdoing, whereas providing them with beneficial medical treatments rewards it, thus undermining the retributive effect of criminal justice. Yet incarcerated offenders are already being punished for their offence by being detained in a correctional facility. According to retributive theories of punishment, criminal offenders’ punishment should be proportionate to the gravity of the offence that they have committed (Brooks, 2012; Lippke, 2014). Compelling a prisoner to cease methadone means imposing the further physical and psychological burdens of opiate withdrawal, including a significant risk of suicide and overdose. This arguably equates to ‘double punishment’; if the prison term has been set so as to be proportionate to the gravity of the offender’s crime, adding further burdens will violate the principle of proportionate punishment. Moreover, this argument applies equally to other beneficial treatments provided to prisoners, so would not justify singling out MMT for cessation.
3. Forced methadone detox might be defended by invoking concerns about the fair distribution of healthcare resources. Some might question whether drug offenders should be provided with treatment when resources are scarce, and many non-offending users who might wish to enrol for MMT are unable to do so due to a lack of treatment places. It might be argued that innocent citizens have a stronger claim to MMT resources than culpable offenders. However, resource constraints on MMT could be substantially alleviated if governments (i) desisted from investing large sums of money on ineffective policing campaigns in an attempt to fight the multinational ‘War on Drugs’ and (ii) reformed mandatory sentencing laws that result in enormous costs to the criminal justice system. Further, even if criminal justice services need to be rationed to allow funding for community healthcare, there are stronger candidates for rationing than prison-based MMT: on average, incarceration in the USA costs around USD $29,000 per prisoner per year in 2013, and there is little evidence that this strategy reduces drug use or recidivism rates for drug offenders (Rich et al., 2005; Chandler et al., 2009); in contrast, the average cost of methadone is USD $4000 per prisoner per year, and MMT has proven effectiveness at reducing drug use and criminal recidivism after release (Mattick et al., 2009). As noted above, Rich *et al.* found that continuing MMT in prisons substantially reduced medical and societal costs post-release (Rich et al., 2015). This raises the possibility that the direct economic costs of prison-based MMT are more than offset by its indirect economic benefits.
4. Finally, hard-line anti-drug campaigners may object to MMT whether it is provided inside or outside prisons. They may argue that, rather than investing funds in treatments that simply replace one addiction with another, the goal should be abstinence from all drugs. MMT may provide a form of symptomatic relief, but the goal should be to cure the addiction. However, this argument is out of line with approaches to symptomatic relief taken elsewhere in medicine. In palliative care, for example, clinicians and patients often opt for partial symptomatic relief rather than aiming for a total cure, and this strategy can be reasonable, particularly where attempts to cure are unlikely to succeed. Heroin addiction is arguably just such a case: though forced detox in prisons might arguably seem to represent an ideal circumstance for producing a total cure of heroin addiction, comprehensive studies from China suggest that it rarely produces long-term cessation of drug use (Liu et al., 2006).

## Concluding Thoughts and Questions

We have been arguing the case against forced methadone detox. Withholding medical treatment from criminal offenders is detrimental to prisoner health, to the safety of the general public and to the public purse. Furthermore, it seems inconsistent with the principle of proportionate punishment, the approaches to symptomatic relief taken elsewhere in medicine and policy recommendations set by international health organizations. In light of this, we believe that the US Federal and State legislatures should work to emulate the approach of many other Western countries.

However, our argument does raise a number of questions that warrant further discussion:

1. Do convicted criminals forfeit the right to receive certain types of healthcare, and even if not, might there be a case for deprioritizing criminal offenders when distributing healthcare resources? Prisoners are sometimes thought to forfeit certain rights that are normally very robust (for example, the rights to freedom of movement and association, as well as the right to political participation). Moreover, in the case of extreme resource scarcity, as with organ transplants, there has been debate as to whether criminal offenders have forfeited some of their rights to costly treatments (Kolata, 1994; Schneiderman and Jecker, 1996). Indeed, some have argued that even non-offenders may forfeit their rights to certain kinds of healthcare; for example, in the literature on personal responsibility for health, some argue that smokers have forfeited their rights to lung cancer treatments and alcoholics to liver transplants (Denier, 2005). We have argued that MMT in prisons could be funded without diverting resources from other goals; however, we recognize that more expensive or less effective treatments might raise genuine quandaries regarding the fair distribution of healthcare resources.
2. How should the goal of crime prevention be accommodated in the setting of public health policy? Traditionally, anti-recidivist measures have been considered outside of the sphere of public health. However, given that crime often results in physical injury, it could be argued that this is an arbitrary exclusion. Moreover, given that MMT, among other interventions, confers both benefits in the form of crime prevention *and* benefits falling within the traditional scope of public health and medicine, it is difficult to see how this intervention could be properly assessed without taking both kinds of benefit into account. There is thus a clear need for an ethical framework capable of doing just that.
3. Relatedly, how, as a practical matter, can the differing values of criminal justice and healthcare be productively reconciled? Delivering MMT and other medical treatments in a criminal justice setting requires cooperation and coordination between two very different cultures: one emphasizes social control and the meting out of deserved punishment, whilst the other focuses on individual autonomy and well-being and (perhaps to a lesser extent) the common good. As we have seen, healthcare can be seen as inimical to retributive goals. Conversely, the coercive measures of criminal justice are often viewed as damaging and disruptive to the therapeutic process (Farabee and Leukefeld, 2001; Chandler et al., 2009). We hold that the goals of punishment do not compete with the goal of providing adequate healthcare for prisoners, particularly in the case of MMT. But the challenge is how we prevent perceived tensions from impeding the realization of both healthcare and correctional goals, and, ultimately, the implementation of MMT in the US prisons?

## Funding

T.D. received funding from the Uehiro Foundation on Ethics and Education and the Wellcome Trust (grant number 100705/Z/12/Z). J.P. received funding from the Wellcome Trust (grant number 100705/Z/12/Z).

## Footnotes

1 Drug-free alternatives included placebo medication, psychosocial counselling, detoxification and wait list control.
